# Effects of Motivational Downshifts on Specific Pavlovian-Instrumental Transfer in Rats

**DOI:** 10.1093/ijnp/pyab075

**Published:** 2022-01-19

**Authors:** Susanne Sommer, Alexandra Münster, Jean-Alain Fehrentz, Wolfgang Hauber

**Affiliations:** 1 Systems Neurobiology Research Unit, University of Stuttgart, Stuttgart, Germany; 2 Department of Neurobiology, University of Stuttgart, Stuttgart, Germany; 3 IBMM, University Montpellier, CNRS, ENSCM, Faculty of Pharmacy, Montpellier, France

**Keywords:** ghrelin, outcome devaluation, outcome-selective Pavlovian-instrumental transfer, rat, satiety

## Abstract

**Background:**

Pavlovian stimuli predictive of appetitive outcomes can exert a powerful influence on the selection and initiation of action, a phenomenon termed outcome-selective Pavlovian-instrumental transfer (sPIT). Rodent studies suggest that sPIT is insensitive to motivational downshift induced by outcome devaluation, an effect that is, however, relatively underexplored.

**Methods:**

Here we examined in detail the effects of distinct shifts in motivation from hunger to a state of relative satiety on sPIT in rats.

**Results:**

A motivational downshift by outcome-specific devaluation immediately prior to testing markedly reduced overall lever responding and magazine entries but left intact the sPIT effect. A motivational downshift prior testing by (1) giving ad libitum rather than restricted access to maintenance diet in the home cage for 24 hours or by (2) a systemic blockade of hormone secretagogue receptor subtype 1A receptors to inhibit orexigenic actions of ghrelin both reduced overall lever responding and magazine entries. Moreover, these latter motivational downshifts reduced the sPIT effect; however, the sizes of the sPIT effects were still large.

**Conclusions:**

Collectively, our rodent findings indicate that major effects of various motivational downshifts are overall inhibition of lever pressing and magazine approach, possibly reflecting reduced general motivation. The observed effects of motivational downshifts on sPIT have implications with regard to the role of general motivating effects in sPIT and to the contribution of Pavlovian-instrumental interactions to excessive food seeking as well as obesity in humans.

**Significance Statement**

Pavlovian stimuli predictive of appetitive outcomes can exert a powerful influence on action selection. Here we examined in rats whether distinct motivational downshifts resulting in satiety alter the ability of food-predictive Pavlovian stimuli to influence action selection. Results show that pre-feeding of the expected outcome immediately prior testing, giving free rather than restricted access to maintenance diet for 24 hours prior to testing, or administering a drug that inhibits actions of the “hunger” hormone ghrelin largely left intact the ability of Pavlovian stimuli to guide action selection but markedly reduced overall lever pressing for and conditioned approach to expected reward. Thus, motivational downshifts do not predominantly reduce the impact of Pavlovian stimuli predictive of appetitive outcomes on action selection but markedly suppress reward-directed activity. Recent findings indicate that food-predictive stimuli prevalent in environments can override satiety effects and promote overeating and obesity. However, our data suggest that satiety can markedly reduce the level of stimulus-induced reward-directed instrumental activity thereby limiting food intake.

## Introduction

Pavlovian stimuli predictive of appetitive outcomes can exert a powerful influence on the selection and initiation of action (Estes 1943; Lovibond 1983; Colwill and Rescorla 1988). For instance, Pavlovian stimuli are able to selectively enhance those actions with which they share an outcome, for example, sucrose-predictive stimuli increase responses associated with sucrose more than responses that earn different outcomes (Rescorla and Solomon 1967; Trapold and Overmier 1972). This phenomenon, termed outcome-selective Pavlovian-instrumental transfer (sPIT), demonstrates that Pavlovian stimuli can critically influence decisions on which course of action to choose. The associative underpinnings of transfer effects in humans and animals are the subject of various theories (Cartoni et al., 2016; Mahlberg et al., 2021). One account suggests that presentation of a stimulus promotes retrieval of the sensory-specific features of the associated outcome, which in turn inclines action selection by promoting the action that leads to that outcome (Ostlund and Balleine 2007).

Importantly, in rats, sPIT seems insensitive to motivational downshift induced by outcome devaluation; that is, pre-feeding with outcomes immediately prior to testing did not affect the magnitude of the transfer effect in sPIT (Holland 2004). This finding is consistent with the idea that sensory-specific features rather than values of the associated outcomes drive sPIT. In line with these findings in rodents, food-associated stimuli can powerfully stimulate eating in sated children and adult humans (Birch et al., 1989; Cornell et al., 1989). Moreover, in humans, satiation failed to reduce stimulus-induced food-seeking in sPIT (Watson et al., 2014). Thus, Pavlovian-instrumental interactions may provide a mechanism through which food-predictive stimuli promote excessive food-seeking and contribute to obesity (Watkins and Olverman 1987; Johnson 2013).

However, the effects of outcome devaluation on sPIT are underexplored and the effect sizes vary considerably across studies, in part due to subtle methodological discrepancies (Cartoni et al., 2016). Moreover, the observed insensitivity of sPIT to outcome devaluation is intriguing in view of findings that Pavlovian and instrumental responding of animals as such remained sensitive to outcome devaluation (Lex and Hauber 2010b), even if similar Pavolvian and instrumental training protocols to those employed for sPIT training are used and result in an insensitivity of sPIT to outcome devaluation. Moreover, tracking and updating of the actual outcome values associated with stimuli and actions is expensive but essential to advantageous action selection. Thus, an insensitivity of sPIT to outcome devaluation can be maladaptive.

Hence, the current study seeks to analyze in detail the effects of motivational downshift on sPIT. Specifically, we examined effects of distinct shifts in motivation from hunger to a state of relative satiety on sPIT. In Experiment 1, we tested the effects outcome-specific devaluation immediately prior to testing on sPIT. In Experiment 2, we examined the effects of giving free rather than restricted access to maintenance diet in the home cage for 24 hours prior to sPIT testing. Experiment 3 tested whether sPIT was influenced by a systemic blockade of GHSR-1A receptors, a pharmacological manipulation that suppresses feelings of hunger mediated by enhanced ghrelin levels (Patterson et al., 2013). Given the key role of the nucleus accumbens (Acb) in sPIT (Cartoni et al., 2016), we also evaluated whether blockade of GHS-R1A altered c-fos levels, a marker of neuronal activation, in the Acb core and shell subregion. Our data reveal that these distinctive motivational downshifts largely left intact the transfer effect in sPIT but significantly decreased general lever-pressing activity as well as conditional approach.

## MATERIALS AND METHODS

All animal experiments were performed according to the European Guidelines of Animal Care and Use for Experimental Procedures (2010/63/EU) and approved by the local authorities (Regierungspraesidium Stuttgart).

### Subjects and Apparatus

Male Sprague Dawley rats (Janvier, St. Berthevin, France) served as subjects in all experiments. The rats were housed in groups of 4 in transparent plastic cages (55 × 39 × 27 cm; Ferplast, Nuernberg, Germany) in a temperature- and humidity-controlled room (20 ± 2°C, 50%–60%) on a 12-hour-light/-dark cycle (lights on at 7:00 am). Throughout the experiments, rats had ad libitum access to water. Standard laboratory maintenance chow (Altromin, Lage, Germany) was given ad libitum for 2 days after arrival; thereafter, food was restricted to 15 g/d per animal.

Training and testing took place in identical operant chambers (24 × 21 × 30 cm; Med Associates, St Albans, VT, USA) housed within sound-attenuating cubicles. Each operant chamber was equipped with a pellet dispenser that delivered 45-mg grain-based pellets (MLab Rodent Test diet, Sandown Scientific, Hampton, Middlesex, UK) into a dual pellet/liquid cup receptacle, which was positioned in the middle of the wall, and a syringe pump that delivered 0.1 mL of a 20% sucrose solution into the same receptacle. Each chamber also contained 2 retractable levers located on either side of the dual cup receptacle. A 24 V⁄3 W house light mounted on the top center of the opposite wall illuminated the chambers. The speaker that delivered the auditory-conditioned stimuli was mounted on the wall opposite to the levers and the receptacle. Two auditory stimuli (white noise [N] and a clicker [C], 80-dB sound pressure level each) served as conditioned stimuli. A computer system (MedPC Software, Med Associates) controlled the equipment and recorded the data.

### Experiment 1: Effect of Outcome-Specific Devaluation on sPIT

**Subjects—**The same animals (n = 23) were used in Experiments 1 and 2.

**Magazine Training—**All subjects received 1 session of magazine training to habituate the animals to the operant chamber. During the magazine training, food pellets (45-mg pellets; formula A⁄I; Sandown Scientific) were delivered on a RT 30-second schedule with no lever available.

**Instrumental Training—**First, rats were trained to press the left and right lever for 2 days using a continuous reinforcement schedule on both levers (Table 1). Then, the rats were trained on the levers each reinforced on a random ratio (RR) schedule. Each lever was trained separately and earned 1 of 2 possible outcomes: pellets or sucrose solution. Any given animal earned both of these outcomes, one pressing the left lever, the other by pressing the right lever. These assignments were counterbalanced across animals. The animals received 4 days of training on the RR-5 schedule (i.e., each action delivered an outcome with a probability of 0.2). Thereafter, animals were shifted to a RR-10 schedule (i.e., each action delivered an outcome with a probability of 0.1) for another 2 days. The animals received 2 sessions each day, 1 with each action–outcome pair, for 30 minutes. The animals had a break of at least 3 hours between sessions, and the order of sessions alternated every day.

**Table 1.** Schematic of behavioral training and testing in Experiment 1 and 2.

Magazine Training	Instrumental Training	Pavlovian Training	sPIT “baseline”	Retraining	sPIT outcome devaluation	Retraining	sPIT outcome devaluation	Retraining	sPIT lab chow *ad lib* vs. restricted	Retraining	sPIT lab *chowad lib* vs. restricted
	A1→O1 A2 → O2	CS1→O1 CS2→O2	CS1: A1 vs. A2 CS2: A1 vs. A2		Day 1: O1, O2 / X CS1: A1 vs. A2 CS2: A1 vs. A2		Day 2: O1, O2 / X CS1: A1 vs. A2 CS2: A1 vs. A2		Day 1: ad.lib, restricted / X CS1: A1 vs. A2 CS2: A1 vs. A2		Day 2: *ad.lib*, restricted / X CS1: A1 vs. A2 CS2: A1 vs. A2

A…action, O…outcome, CS…conditioned stimulus, X…counterbalanced.

**Pavlovian Training—**Thereafter, rats received 8 sessions of Pavlovian conditioning. Two 80-dB SPL auditory stimuli (white noise and a clicker) served as conditioned stimulus (CS+) and were paired with either pellets or 20% sucrose solution in a counterbalanced manner. Four presentations of each stimulus were given in each session interspersed with variable inter-stimulus interval (ISI) (90–270 seconds) in which no stimuli were presented. The stimuli presentations were 2 minutes long, during which the appropriate outcome was delivered on an RT 30-second schedule of reinforcement. There were 2 blocks of interval combinations (CINI and NICI, noise-ISI-clicker-ISI), which were presented in random order. Each block was presented twice so that every stimulus was presented 4 times. The Pavlovian training sessions were approximately 45 minutes in duration.

Thereafter, animals received instrumental training as a reminder. Two separate sessions were given, one for each lever, using an RR-20 schedule (i.e., each action delivered an outcome with a probability of 0.05) for an additional day.

**Transfer Test—**Following the last day of instrumental training, an initial transfer test (“baseline”) was performed with animals maintained under restricted feeding conditions as described above (and without prior outcome devaluation) (Table 1). In this and all subsequent transfer tests, both levers were always p one resent throughout the session, and lever presses were not reinforced. Also, in this and all subsequent transfer tests responding on both levers was extinguished at the beginning of the transfer for 6 minutes to reduce baseline performance (not recorded). Then, after 6 minutes elapsed, each CS+ was presented 4 times over the next 32 minutes in the following order: CINI NICI NICI CINI. Each CS+ lasted 2 minutes and had a fixed ISI of 2 minutes. Magazine entries and lever-pressing rates were recorded throughout the session. The transfer test was based on a protocol by Corbit and Balleine (2003).

**Transfer Test After Outcome Devaluation—**Thereafter, we tested sPIT after outcome devaluation using a within-subject cross-over design in which 2 transfer tests were performed: in the first test, sucrose reward was devalued in one-half of the animals and pellet reward in the other one-half. In the second test, the reverse assignment was used (Table 1).

Specifically, all animals first received 2 days of Pavlovian retraining with 1 session per day as described above. On the next day, all animals received instrumental re-training on 2 sessions under RR-20 for 1 day as described above. Then, the first transfer test was performed after devaluation by pre-feeding. To this end, transparent plastic cages (42 × 26 × 18 cm, Ferplast) were used for pre-feeding. Prior to transfer testing, all animals were given a 40-minute ad libitum access to 1 of the 2 outcomes in the feeding cages (1 animal per cage). One-half of the animals received pellets (in a glass bowl), and the other one-half received the sucrose solution (in a drinking bottle). Immediately after pre-feeding, the rats were placed into the operant chambers and the transfer test was conducted. On the next days, the rats received between Pavlovian retraining for 2 days and instrumental retraining for 1 day (2 sessions, for each lever) as described above. Thereafter, rats were given a second transfer test 7 days later after outcome devaluation using the opposite assignment.

### Experiment 2: sPIT and Effects of a Motivational Downshift Induced by ad libitum Lab Chow

Thereafter, we tested sPIT under a restricted vs ad libitum feeding regimen using a within-subject cross-over design (Table 1). Initially, all animals received 3 days of Pavlovian retraining with 1 session per day as described above. On the next day, all animals received instrumental re-training on 2 sessions under RR-20 for 2 days as described above. Then, the first transfer test was performed. One-half of the rats received ad libitum laboratory chow in their home cages for 24 hours prior to testing, and the other one-half was maintained on the regular restricted laboratory chow regimen (15 g/d). On the next days, the rats received in-between Pavlovian and instrumental retraining, after which they were given a second transfer test with ad libitum laboratory chow for 24 hours prior testing for the other one-half of the rats.

### Experiment 3: sPIT and Effects of a Motivational Downshift Induced by a Ghrelin Antagonist—Role of the Nucleus Accumbens

**Subjects—**Another group of animals (n = 23) was used in Experiment 3 and subjected to magazine, instrumental, and Pavlovian training as described for Experiment 1.

**Drugs—**To analyze an involvement of GHSR-1A, the effects of JMV2959 (Moulin et al., 2013) (5 and 10 mg/kg, i.p.; IBMM, Univ Montpellier, CNRS, ENSCM, Faculty of Pharmacy, Montpellier, France) on sPIT were tested. JMV 2959 at the doses used blocks GHSR-1A receptors, but does not bind to dopamine D1 or D2 receptors, and has no effect on locomotor activity or gross behavior (Jerlhag et al., 2009, 2010, Jerlhag and Engel 2011). JMV2959 was dissolved in vehicle (0.9% NaCl) and administered 20 minutes prior to test.

**Transfer Tests After Drug Administration—**Drug effects on sPIT were assessed in 2 separate experiments using a within-subjects design, respectively. That is, in the first transfer test, 1 subgroup of the animals received drug, and the other subgroup received vehicle. The reverse assignment was used in the second transfer test. In Experiment 3A, JMV2959 at a dose of 5 mg/kg i.p. was tested in n = 23 rats; in Experiment 3B, JMV2959 at a dose of 10 mg/kg i.p. was tested in a subgroup of n = 12 only due to limited drug availability. To assess drug effects further, individual laboratory chow intake was monitored in a consumption test for 1 hour immediately after transfer testing in a transparent plastic cage with a dish containing standard laboratory chow (1 animal per cage; 425 × 265 × 150 mm; Ferplast).

In a subsequent Experiment 3C, drug effects (JMV 10 mg/kg, i.p.) on c-fos expression in the Acb was assessed using a between-subjects design using all animals (n = 23). That is, 1 subgroup of the animals (n = 11) received JMV 2959, and the other subgroup (n = 12) received vehicle; 80 minutes after drug administration, animals were killed for immunohistochemical analysis.

### Histology

Animals were killed and perfused transcardially with 0.01% heparin sodium salt in phosphate-buffered saline (PBS), followed by 4% paraformaldehyde in PBS. The brains were removed, postfixed in paraformaldehyde for 24 hours, and dehydrated in 30% sucrose for at least 48 hours. Coronal prefrontal brain sections (35 μm) were made and washed for 10 minutes in PBS followed by a 2-hour incubation in a PBS blocking solution (1× PBS, 10% normal goat serum, 0.5% Triton X-100). Sections were then incubated for 36 hours at 4°C in the primary antibody PBS solution containing rabbit anti-c-Fos (1:6000; MCA-2H2, EnCor Biotechnology Inc.), 2% normal goat serum, and 0.2% Triton X-100. Sections were washed 4 times for 10 minutes in PBS before being mounted on slides before being coverslipped with DAKO Fluorescent mounting medium.

### Fluorescent Imaging and Quantification of Immunoreactive Cells

Five images per hemisphere within the Acb were taken and evaluated from each rat using an Olympus AX70 Axiocam 503. Neurons expressing c-Fos were counted by an observer blind to the experimental conditions. For each animal, the mean number of c-Fos–positive cells in all images was calculated and scaled to c-Fos–positive cells/mm².

### Data Analysis and Statistics

Data were given as means ± SEM throughout the paper. Data were subjected to repeated-measures ANOVA. To specify stimulus effects on responding, ANOVA was followed by planned comparisons on lever presses during ISI vs “same” and vs “diff” as well “same” vs “diff” (the latter being a main criterion of sPIT). All statistical computations were performed with STATISTICA (Tibco, Vers. 13.3). The level of statistical significance (α-level) was set at *P* ≤ .05; α-levels *P* > .05 were designated as n.s. (not significant).

In addition, the effect size *r* (Pearson’s r) of stimulus effects on lever pressing, that is, for lever presses during ISI vs “same” and “diff” and “same” vs “diff,” were calculated. Each calculation was based on the F-value of a planned comparison of lever presses across ISI/stimuli using the following equation (Field, 2013):


$$\begin{array}{l} r=\sqrt{\frac{F(1,df_{R})}{F\left(1,df_{R}\right)+df_{R}}} \end{array}$$

## RESULTS

### Experiment 1: sPIT and Effects of Outcome-Specific Devaluation

**Figure 1.**
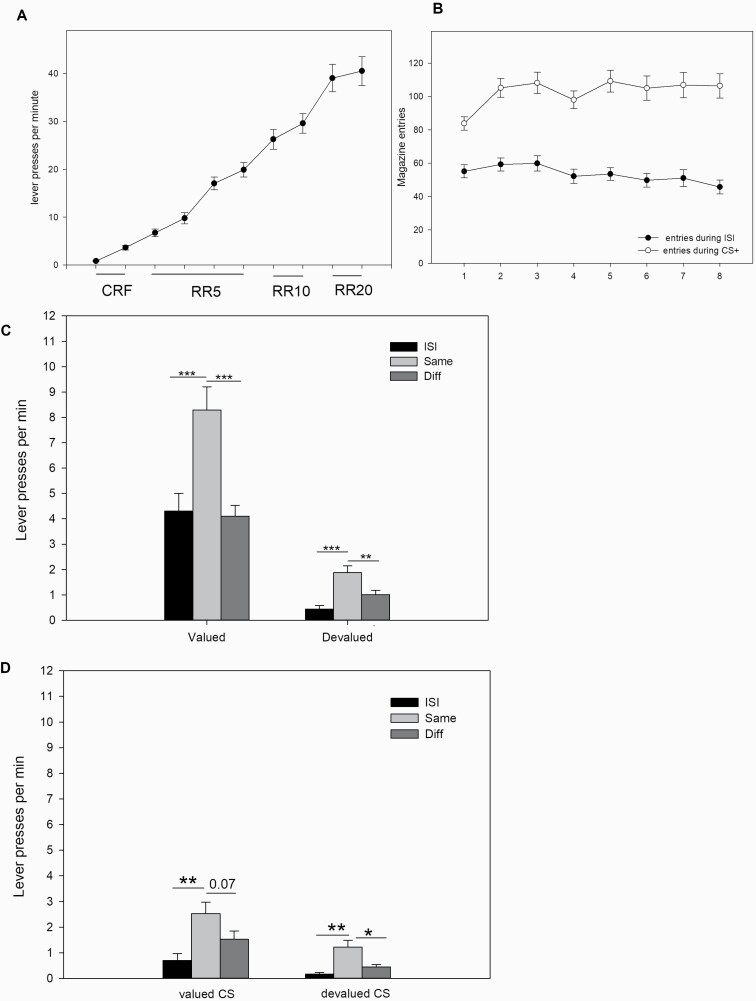
(A) Mean LP per minute (±SEM) per session over 10 instrumental training days with different schedules of reinforcement (continuous reinforcement schedule, RR-5, RR10, RR-20). (B) Mean magazine entries (±SEM) during stimulus presentation intervals (noise/click) or interstimulus intervals per session over 8 Pavlovian training days. Data are form n = 23 animals. (C) Mean LP per minute (±SEM) during the sPIT test with (“devalued”) or without (“valued”) prior outcome specific devaluation (n = 23). Note that sPIT data with prior outcome devaluation are averaged across 2 separate transfer tests with cross-over devaluation of 1 outcome. *P < .05; **P < .01; ***P < .001, ANOVA followed by planned comparisons. (D) Lever presses (mean LP per minute ± SEM) across stimuli after outcome devaluation. Data given in the right graph in Figure 1C are re-plotted to show effects of outcome devaluation in detail. In each transfer test under devaluation, only 1 of 2 outcomes was devalued, the other was valued. In this figure, the effects of the devalued stimulus (“devalued CS”) on pressing the devalued lever (Same) or valued lever (diff) vs the effects of the valued stimulus (“valued CS”) on pressing the valued lever (same) or the devalued lever (diff) are shown. For more details see text. *P* = .07; *P < .05; **P < .01; ***P < .001, ANOVA followed by planned comparisons.

First, animals underwent instrumental and Pavlovian training; lever press rates and magazine entries from all animals (n = 23) are given in Figure 1A and B. In the transfer test, animals without prior outcome-specific devaluation displayed a robust sPIT effect (Fig. 1C, left figure), that is, a specific increase of responding on the lever that had been paired during training with the same outcome as the respective stimulus ("same" > "diff"). A sPIT effect was also apparent after outcome specific devaluation (Fig. 1C, right figure). An ANOVA across sPIT data from the test with and without outcome devaluation revealed an effect of stimulus identity (ISI, "same", "diff") (F[2, 44] = 84.1, *P* < .001), value (outcome valued, devalued) (F[1, 22] = 19.9, *P* < .001), and a stimulus identity × value interaction (F[2, 44] = 7.4, *P* < .01). Note that lever press rates during ISI, "same", and "diff" from the initial transfer test represent means from 1 session, and lever press rates from sessions with prior outcome devaluation are means from 2 separate sessions with cross-over devaluation of 1 outcome, respectively. Planned comparisons confirmed elevated response rates during CS+ on the "same" lever compared with response rates during ISI ("same" > ISI) as well on the different lever ("same" > "diff") both in the valued and devalued condition. The stimulus identity × value interaction indicates that outcome devaluation reduced the magnitude of the sPIT effect. Nevertheless, an effect size analysis (Table 2) revealed that the sPIT effect after outcome devaluation is still large.

**Table 2.** Effects sizes (Pearson’s *r*) in the transfer tests in Experiments 1, 2, and 3B from all contrasts

Experiment	Contrast/condition	Valued	Devalued
Experiment 1	Baseline-same	0.68	0.84
	Baseline-diff	0.05	0.53
	Same-diff	0.67	0.52
Experiment 2	Baseline-same	0.58	0.51
	Baseline-diff	0.51	0.36
	Same-diff	0.58	0.62
Experiment 3B	Baseline-same	0.68	0.50
	Baseline-diff	0.16	0.22
	Same-diff	0.63	0.47

Calculations are based on F-values of respective contrast analysis. On a non-linear scale, *r* ≥ 0.1 denote small effects, *r* ≥ 0.3 medium effects, and *r* ≥ 0.5 large effects (Field, 2013). Diff, different.

Lowered sPIT effects across tests in Experiment 1 could be due to repeated testing with decreasing lever press performance. However, this is unlikely because interim Pavlovian and instrumental training has been given. Accordingly, across both transfer tests with devaluation, there is a significant effect of stimulus identity (ISI, "same", "diff") (F[2, 44] = 19.2, *P* < .01), but not of day (devaluation day 1, devaluation day 2) (F[1, 22] = 0.42, n.s.) and there is no stimulus identity × day interaction (F[2, 44] = 0.86, n.s.).

Results further show the baseline magazine entries per minute (valued: 2.67 ± 0.32; devalued: 0.75 ± 0.10) and overall magazine entries per minute during presentation of both stimuli (valued: 38.54 ± 14.0; devalued: 17.40 ± 1.73) markedly differed in animals with and without outcome devaluation. An ANOVA revealed an effect of period (baseline, click/noise presentation) (F[1, 22] = 277,5, *P* < .001) and value (outcome valued, devalued) (F[1, 22] = 39.9, *P* < .001) as well as a period × value interaction (F[1, 22] = 32.1, *P* < .001).

In a subsequent analysis, we separately assessed the effects of outcome devaluation with pellets vs sucrose on sPIT performance during ISI and in the “same” condition. Note that in any sPIT test, the 2 levers that were associated with either sucrose or pellets during training were always available, and stimuli predictive for sucrose and pellets during training were presented in a random order. Thus, the “same” condition always includes 2 sub-conditions that assessed (1) effects of the sucrose-predictive stimulus on pressing the lever associated with sucrose during training, and (2) effects of the pellet-predictive stimulus on pressing the lever associated with pellets during training. By contrast, the “diff” condition assessed effects of the sucrose-predictive stimulus on pressing the lever associated with pellets and effects of the pellet-predictive stimulus on pressing the lever associated with sucrose.

Because devaluation of both outcomes (sucrose or pellets) was performed in separate devaluation transfer tests, we can compare in each transfer test the effects of the devalued outcome (say sucrose) on both “same” sub-conditions (sucrose stimulus effects on sucrose lever [devalued] vs pellet stimulus on pellet lever [valued]) using within-subject comparisons. Likewise, in each devaluation transfer test, we can compare effects of the devalued outcome (say sucrose) on pressing the lever for the devalued (sucrose) vs valued (pellets) reward during ISI (baseline responding). For instance, for rat #1, the right lever yielded sucrose, the left lever pellets during training; the noise stimulus indicated sucrose, the click stimulus pellets during training (Table 3). On day 1, pellets were devaluated for this animal. When the noise stimulus for sucrose was presented in the sPIT session, the animal could press either the lever that earned sucrose ("same") or pellets ("diff") during training. When the click stimulus for pellets was presented in this same sPIT session, the animal could press either the lever that earned pellets ("same") or sucrose ("diff") during training. Moreover, during ISI, rat #1 could press the lever that either earned sucrose or pellets during training. Because pellets were devalued on day 1 for this subject, we expected that in this devaluation transfer test, during ISI the rat would prefer the right lever for sucrose over the left lever for pellets. Furthermore, due to pellet devaluation, we expected that pressing of the right lever for sucrose would be higher in this transfer test if the noise stimulus that predicts sucrose (valued “same” condition) is presented compared with pressing the left lever for pellets if the click stimulus is given that predicts pellets (devalued “same” condition). Note that in the “diff” condition, a lever that predicts devalued reward is chosen during presentation of a stimulus that predicts a valued outcome (or vice versa) which, in view of value disparities, represents a more complex situation. On day 2, sucrose was devalued, thus creating an inverse situation. To analyze responding during ISI, "same" and "diff" undervalued, and devalued conditions , we used, for example, for rat #1 averaged values from day 1 and day 2 from the valued same/diff condition (Table 3, mean of Y1, Y4; Z1, Z4) and respective ISI (means of X1, X4) and from the devalued same/diff condition (Table 3, mean of Y2, Y3; Z2, Z3) and the respective ISI (mean of X2 and X3).

**Table 3.** Example data for rat #1.

Animal ID	Day	Devalued reward	Lever/position	Lever/reward	Stimulus/identity	Stimulus/Reward	ISI	Same lever presses/min	Diff lever presses/min
1	1	Pellet	Right	Sucrose	Noise	Sucrose	X1	Y1	Z1
1	1	Pellets	Left	Pellet	Click	Pellet	X2	Y2	Z2
1	2	Sucrose	Right	Sucrose	Noise	Sucrose	X3	Y3	Z3
1	2	Sucrose	Left	Pellet	Click	Pellet	X4	Y4	Z4

Lever position-reward and stimulus identity-reward assignments used for rat # 1. Sucrose solution and pellet served as rewards, click and noise as stimuli. Lever presses/min during sPIT (denoted here as X 1-4, Y 1-4, Z 1-4) were recorded under ISI, “same” and “diff” conditions for test day 1 (devaluation of pellets) and test day 2 (devaluation of sucrose solution). For calculations on lever press values see text. ID, identity code.

Diff, different.

As shown in Figure 1D, during ISI, pressing rates for the lever associated with valued outcome was higher compared with the lever associated with the devalued outcome. Likewise, the “same” stimulus elevated performance of the action delivering the outcome predicted by the CS to a higher degree if this particular outcome was valuated. In line with this description, an ANOVA revealed an effect of stimulus identity (ISI, "same", "diff") (F[1, 22] = 21,33 *P* < .001) and value (valued, devalued) (F[2, 44] = 11,65, *P* < .001) but no stimulus identity × value interaction (F[2, 44] = 1.16, n.s.). Planned comparisons confirmed elevated response rates during CS+ on the same lever compared with response rates during ISI ("same" > ISI) as well on the different lever ("same" > "diff") in both the valued and devalued condition (note that the valued same > diff contrast tended to reach significance, *P* = .07). The significant value effect highlights that the sPIT performance under devalued compared with valued conditions was markedly lower. However, the stimulus identity × value interaction clearly missed significance, suggesting that a sPIT effect as such was still evident under devalued conditions. An analysis of magazine entries further revealed that, after outcome devaluation, responses per minute to the food port were higher during presentation of the valued stimulus (10.04 ± 1.18) compared with the devalued stimulus (7.35 ± 0.67) (F[1,22] = 10.3, *P* < .01).

### Experiment 2: sPIT and Effects of a Motivational Downshift Induced by Ad Libitum Lab Chow

**Figure 2.**
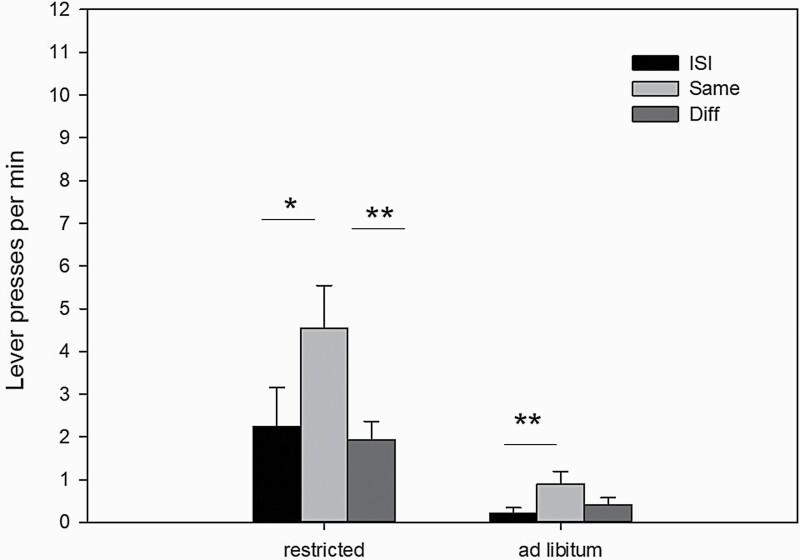
Mean lever presses per minute (±SEM) during the sPIT test after restricted or ad libitum laboratory chow feeding (n = 23). *P < .05; **P < .01; ANOVA followed by planned comparisons.

The same animals (n = 23) were then tested under restricted vs ad libitum laboratory chow feeding regimens (Fig. 2). An ANOVA revealed an effect of stimulus identity (ISI, "same", "diff") F[1, 22] = 18.0, *P* < .01), feeding regimen (ad libitum, restricted) (F[2, 44] = 9.7, *P* < .001), and stimulus identity × feeding regimen interaction (F[2, 44] = 9.4, *P* < .001). Planned comparisons confirmed elevated response rates during CS+ on the same lever compared with response rates during ISI ("same" > ISI) both in the ad libitum and restricted condition. Moreover, a sPIT effect indicated by elevated response rates was observed during CS+ on the "same" lever compared with response rates on the different lever ("same" > "diff") after restricted but not after ad libitum feeding. However, an effect size analysis (Table 2) revealed that the sPIT effect after ad libitum feeding was still large.

In a subsequent analysis, we separately assessed the effects of ad libitum pre-feeding on the “same” conditions. Results demonstrate (data not shown) that lever pressing in both sub-conditions (sucrose-predictive stimulus promotes pressing the lever associated with sucrose during training; pellet-predictive stimulus promotes pressing the lever associated with pellets during training) was reduced to a similar extent (t_22_ = 0.56, n.s.). In other words, ad libitum pre-feeding did not selectively influence sPIT performance.

The feeding regimen also influenced baseline magazine entries per minute (ad libitum: 0.8 ± 0.16; restricted: 2.53 ± 0.53) and total magazine entries/min during presentation of both stimuli (ad libitum: 13.64 ± 1.29; restricted: 30.7 ± 2.15). Accordingly, an ANOVA revealed an effect of period (ISI, click/noise presentation) (F[1, 22] = 118.2, *P* < .001) and feeding regimen (ad libitum, restricted) (F[1, 22] = 182.1, *P* < .001) and a period × feeding regimen interaction (F[1, 22] = 123.9, *P* < .001). Together, these data suggest that prior ad libitum feeding had inhibitory effects on sPIT, that is, reduced the sPIT effect as well as overall lever pressing activity and conditioned approach responses to the magazine.

### Experiment 3: sPIT and Effects of a Motivational Downshift Induced by a Ghrelin Antagonist—Role of the Acb

On the final day of Pavlovian training, animals (n = 23) made an average of 103.2 ± 6.6 magazine entries per session during CS + presentations compared with 47.1 ± 3.4 magazine entries without CS + presentations (ISI). On the final day of instrumental training, the rats made an average of 59.3 ± 3.1 lever presses per minute per session. Then, the effects of low-dose (n = 23) and high-dose (n = 12) JMV 2959 on sPIT were tested in separate experiments and, due to sample size disparities, analyzed in separate ANOVA.

**Figure 3.**
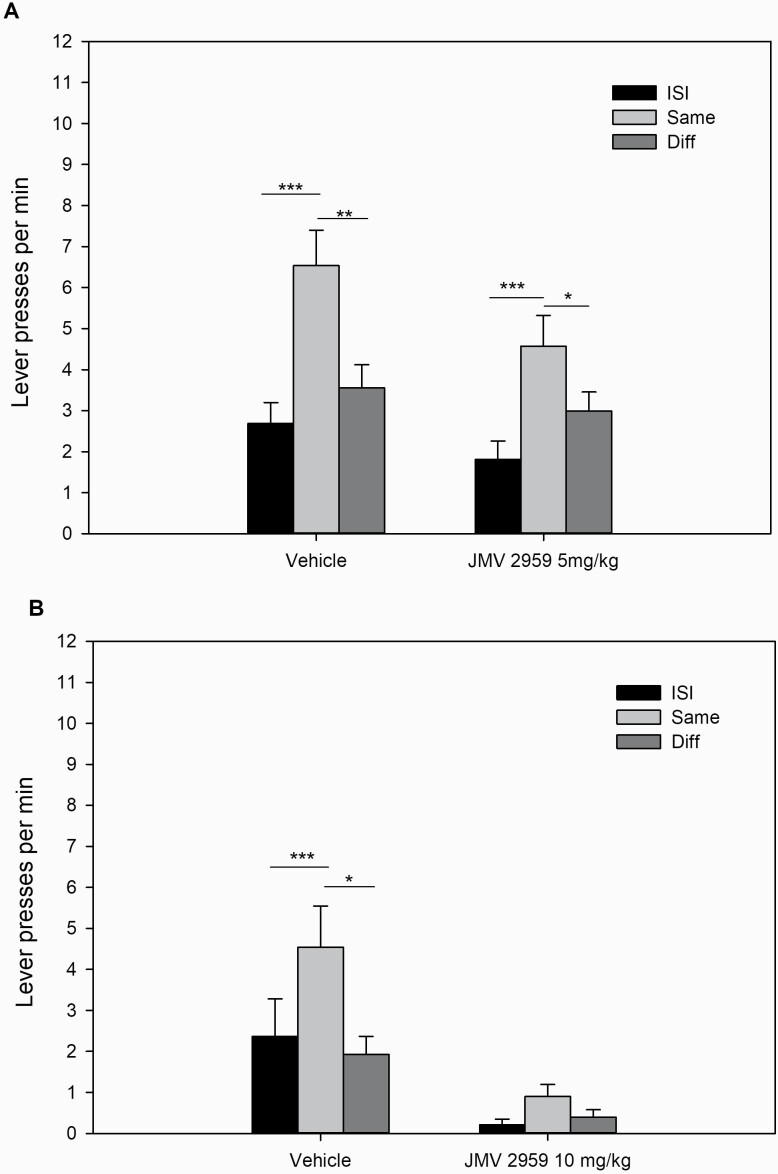
Mean lever presses per minute (±SEM) during the sPIT test after administration of vehicle or the ghrelin antagonist JMV2959. (A) 5 mg/kg, i.p. (n = 23). (B) 10 mg/kg, i.p. (n = 12). *P < .05; **P < .01; ***P < .001; ANOVA followed by planned comparisons.

Experiment 3A demonstrated that after administration of vehicle or the ghrelin antagonist JMV 2959 [5 mg/kg], animals displayed a sPIT effect ("same">"diff") (Fig. 3A). An ANOVA revealed no effect of treatment (vehicle, JMV2959 5 mg/kg) (F[1, 23] = 2.25, n.s.), an effect of stimulus identity (ISI, , "same", "diff") (F(2, 46) = 20.4, *P* < .001), and no stimulus identity × treatment interaction (F[2, 46] = 1.86, n.s.). Planned comparisons confirmed elevated response rates during CS+ on the "same" lever compared with response rates during ISI ("same" > ISI) and on the "same" lever ("same" > "diff") after vehicle and higher response rates during CS+ on the "same" lever compared with response rates during ISI ("same"> ISI) both under vehicle and JMV2959. A subsequent consumption test after transfer testing revealed reduced laboratory chow consumption after administration of JMV 2959 (9.7 ± 0.4 g) relative to vehicle (11.4 ± 0.3 g) (t_23 _= 3.20, *P* < .01).

Further analysis revealed that baseline magazine entries per minute (vehicle 3.60 ± 0.58; JMV2959 5 mg/kg: 2.99 ± 0.68) and total magazine entries per minute during presentation of both stimuli (vehicle: 41.1 ± 2.48; JMV2959 5 mg/kg: 31.79 ± 2.53) differed as a function of treatment. An ANOVA revealed an effect of period (ISI, click, noise presentation) (F[1, 23] = 262.9, *P* < .001) and treatment (vehicle, JMV2959) (F[1, 23] = 21.4, *P* < .001) and a period × feeding regimen interaction (F[1, 23] = 124.8, *P* < .001). Thus, JMV 2959 at a low dose was able to reduce conditioned magazine approach but not the influence of stimuli on lever selection as well as overall lever press rates.

Experiment 3B revealed that the sPIT effect ("same" > "diff") was markedly lower in animals (n = 12) after administration of the ghrelin antagonist JMV 2959 at a higher dose (10 mg/kg) relative to vehicle administration (Fig. 3B). An ANOVA revealed an effect of stimulus identity (ISI, same, diff) (F[2, 22] = 7.7, *P* < .01) and treatment (vehicle, JMV2959 10 mg/kg) (F[1, 11] = 12.2 *P* < .01) and a near significant stimulus identity × treatment interaction (F[2, 22] = 3.0, *P* = .07). Planned comparisons revealed elevated response rates during CS+ on the "same" lever compared with response rates during ISI ("same" > ISI) and on the "same" lever ("same">"diff") under vehicle treatment only. Nevertheless, an effect size analysis (Table 2) revealed that the sPIT effect after high-dose JMV2959 treatment is still substantial.

Moreover, baseline magazine entries per minute (vehicle 3.60 ± 0.58; JMV2959 10 mg/kg: 2.99 ± 0.68) and total magazine entries per minute during presentation of both stimuli (vehicle: 41.1 ± 2.48; JMV2959 10 mg/kg: 31.79 ± 2.53) differed as a function of treatment. An ANOVA revealed an effect of period (ISI, click, noise presentation) (F[1, 11] = 86.22, *P* < .001) and treatment (vehicle, JMV2959) (F[1, 11] = 28.82, *P* < .001) and a period × feeding regimen interaction (F[1, 11] = 40.05, *P* < .001). Thus, high-dose JMV 2959 treatment reduced the sPIT effect, overall lever pressing, and conditioned approach responses to the magazine. A consumption test after transfer testing showed reduced laboratory chow consumption after administration of JMV 2959 (8.2 ± 0.9 g) relative to vehicle (11.9 ± 0.5 g) (t_11 _= 5.0, *P* < .001).

Then, the effects of JMV 2959 (10 mg/kg) (n = 12) vs vehicle (n = 11) administration on c-fos expression in the Acb were examined using a between-subject design. Results revealed that, relative to vehicle administration, treatment with JMV2959 decreased the number of c-fos–positive cells in the AcbC (*t-*test, t_21 _= 3.31, *P* < .01) but not in the AcbS (t_21 _=_ _0.44, n.s.) (Table 4).

**Table 4.** Number of c-Fos-positive cells (means ± SEM) in the Acb core and shell in rats after administration of vehicle or the ghrelin antagonist JMV 2959 (10 mg/kg, i.p.)

Treatment	Acb corec-fos (counts/mm^2^)	ACB shellc-fos (counts/mm^2^)
Vehicle (n = 11)	88.9 ± 14.2	19.2 ± 5.7
JMV 2959 (n = 12)	38.3 ± 6.7**	22.5 ± 4.9

t-test, Acb: Nucleus accumbens

*t* test, **P < .01.

## Discussion

Here we examined effects of distinct shifts in motivation from hunger to a state of relative satiety on sPIT. Results reveal that a motivational downshift by outcome-specific devaluation immediately prior to testing markedly reduced overall lever responding and magazine entries but left intact the sPIT effect (same > diff) as such. A motivational downshift—by giving free rather than restricted access to maintenance diet in the home cage for 24 hours prior to testing or by a systemic blockade of GHSR-1A receptors by a high dose of JMV 2959—also reduced overall lever pressing and magazine responding and, moreover, reduced the sPIT effect. Though sPIT effects were found to be lowered by ad libitum feeding and high-dose JMV2959 treatment, inspection of effects sizes suggests that sPIT effects were still substantial.

### Outcome-Selective Devaluation and sPIT

Outcome-specific devaluation in Experiment 1 reduced the overall level of lever pressing and magazine entries but did not alter the sPIT effect. One of the few related studies also demonstrated that outcome-specific devaluation reduced baseline responding and left intact the ability of the “same” stimulus to enhance instrumental responding (Rescorla 1994b). However, this study will be not considered further, because discriminative stimuli have been employed that produce markedly different transfer effects than Pavlovian stimuli used in the current study (Rescorla 1994a). A key paper by Holland (2004) found that outcome-specific devaluation significantly attenuated baseline responding and did not alter transfer effect magnitude during presentation of “same” Pavlovian stimuli. Moreover, in this study, devaluation effects on baseline responding interacted with the amount of instrumental training that has been systematically varied. Although in this and our study outcome-specific devaluation had comparable and pronounced suppressive effects on baseline responding, the enhancement of lever pressing during stimulus presentation varies considerably. It is well known that the type of reinforcement schedule used in instrumental training markedly influences the magnitude of devaluation effects. Specifically, ratio schedules are considerably more sensitive than interval schedules to post-conditioning changes in reinforcer value (Dickinson et al., 1983). The fact that Holland (2004) used an interval schedule of instrumental training, whereas we used a ratio schedule, provides, therefore, one explanation for pronounced devaluation-induced suppression of lever-pressing activity seen in our study. Moreover, the amount of Pavlovian and instrumental training varies across both studies, a parameter that also influences sPIT performance (Holmes et al., 2010; Cartoni et al., 2016). In addition, comparative studies (Colwill and Rescorla 1985) revealed that outcome devaluation using an outcome-specific satiety protocol as used here exerts stronger suppressive effects on instrumental responding than a conditioned taste aversion protocol employed by Holland (2004). Thus, unlike an earlier study (Holland 2004), we observed considerable suppression of overall lever-pressing activity after outcome-specific devaluation. We suspect that major procedural differences may predominantly account for discrepant findings. Nevertheless, our study also shows that outcome devaluation did not interfere with the ability of stimuli to bias action selection.

The observation that outcome devaluation reduced magazine entries as well suggests that outcome devaluation had more general effects on reward-directed activity. Critically, a detailed analysis further showed that, for instance, devaluation of pellets not only markedly reduced the ability of the pellet-predictive stimulus to stimulate pellet-predictive lever activity and conditional approach to the magazine but, importantly, also reduced the ability of the sucrose-predictive stimulus to stimulate pressing the sucrose-predictive lever and conditional approach to the magazine, though to a lesser extent (and vice versa). This latter finding highlights that devaluation of one outcome also exerts non-specific inhibitory effects on responding during the transfer test, that is, it reduced lever pressing and magazine entries during presentation of the stimulus that predicts the other, non-devalued outcome.

### Free Access to Lab Chow or Ghrelin Antagonist Administration and sPIT

Experiment 2 revealed that a motivational downshift by 24 hours ad libitum access to laboratory chow significantly reduced baseline responding, overall lever pressing, and magazine entries during stimulus presentation as well as the magnitude of sPIT effect. However, the effect size analysis suggests that the sPIT effect as such is still large. Consistent with this finding, ad libitum access to laboratory chow reduced baseline responding and the transfer effect during presentation of the “same” stimulus, a pattern of effects that, however, was only partially confirmed in another experiment in the same study (Corbit et al., 2007). We cannot exclude that repeated transfer testing in extinction could have contributed to reduced baseline responding across Experiments 1 and 2. However, this possibility seems less likely, because responding did not differ between subsequent transfer tests under devaluation in Experiment 1, probably due to interim Pavlovian and instrumental training.

Moreover, the effect of the motivational downshift induced by ad libitum feeding on sPIT could be influenced by the degree of prior food restriction. The effects of laboratory chow restriction on body weight development can be influenced by various variables (e.g., laboratory chow composition, enrichment, etc.). Therefore, rats maintained under rather disparate restrictive feeding regimens that provide 12 or 17 g/d laboratory chow both resulted in approximately 85% of their free feeding weight and allow for an increase in body weight over time (Youngblood et al. 1997; Allen et al., 2013). Our rats received 15 g/d laboratory chow as well food reinforcement during training and testing, which may result in a similar and moderate reduction of their free feeding body weight. Notwithstanding, in which ways the effect of a motivational downshift by ad libitum feeding on sPIT depends on the degree of prior laboratory chow restriction and free feeding weight is an important open question.

Experiment 3 showed that a blockade of GHSR-1A receptors, a pharmacological manipulation that suppresses feelings of hunger mediated by enhanced ghrelin levels (Patterson et al., 2013), can reduce the sPIT performance. Specifically, the high dose of JMV 2959 reduced instrumental activity and conditional approach and tended to reduce the sPIT effect. As in Experiment 2, the effect size analysis suggests that the sPIT effect as such is still substantial. In light of the fact that JMV2959 reduced food intake in the consumption test performed here, drug effects may be mediated in part by a reduced primary motivation for food. In line with this notion, fasting is known to increase endogenous levels of the “hunger” hormone ghrelin (Bagnasco et al., 2002) and to stimulate chow intake in rats, an effect that was largely prevented by systemic administration of JMV2959 at a similar dose as used here (Schele et al., 2016). Moreover, because JMV2959 can also decrease operant responding for food, blockade of GHS-R1A signaling has been suggested to reduce incentive motivation to work for reward (Skibicka et al., 2012; Sommer and Hauber 2016), an effect that could add to reduced PIT performance seen here. In addition, it is possible that non-specific motor effects could account for reduced responding under JMV2959 seen here. However, JMV2959 in the dose range used had no effect on locomotor activity (Jerlhag et al., 2009, 2010; Jerlhag and Engel 2011). Our results further demonstrate that the blockade of GHS-R1A reduced c-fos levels, a marker of neuronal activation, in the Acb core but not Acb shell. The lack of effect in the Acb shell was unexpected in view of electrophysiological and pharmacological experiments showing that JMV2959 inhibited ghrelin’s effects on neuronal activity and neurotransmission in the Acb shell while having no effects of its own (Vestlund et al., 2019; Sustkova-Fiserova et al., 2020). However, data on JMV2959-induced effects on neuronal activity in the Acb core are, to the best of our knowledge, not available. Overall, our immunohistochemistry data suggest that a JMV2959-induced reduction of Acb core neuronal activity could be at least one mechanism that contributed to inhibitory effects of GHRS-1A. Of course, JMV2959 effects on neuronal activity in other areas that express GHRS-1A receptors and support sPIT such as the ventral tegmental area (Howard et al., 1996; Cartoni et al., 2016) may add to the behavioral effects seen here.

### Stimulus Guidance in sPIT and Implications for Control of Food Intake

Overall, Experiments 1–3 provide evidence for the idea that motivational downshifts exert general inhibitory effects in transfer tests, that is, reduced overall lever-pressing activity and conditional approach responses. With regard to the sPIT effect, results are less consistent: selective outcome devaluation left intact the sPIT effect, inhibition of GHRS-1A tended to reduce the sPIT effect and ad libitum feeding reduced the sPIT effect. Yet, the effect size analysis suggests that substantial sPIT effects are still evident across distinct motivational downshifts.

Theoretical accounts hold that outcome-selective transfer could be mediated both by outcome value representations and representations of sensory aspects of the outcomes (Dickinson and Balleine 2002). Yet, empirical studies showing that the transfer effect in sPIT was insensitive to outcome devaluation (Holland 2004) suggest that the values of outcome representations that may be reduced by outcome devaluation are thought to play only a minor role in sPIT (Dickinson and Balleine 2002; Cartoni et al., 2016). Instead, these findings have been interpreted as evidence that transfer effects in sPIT must be guided by representations of sensory aspects of the outcomes that are deemed to be unaffected by outcome devaluation (Holland 2004).

However, results of our study point to a more complex view. First, our data suggest that a motivational downshift induced by ad libitum feeding is able to reduce the magnitude of the sPIT effect, at least to some extent. Second, our observation that, after outcome-selective devaluation, lever pressing was higher for the valued outcome relative to the devalued outcome during presentation of the “same” stimulus and during ISI is consistent with the idea that representations of sensory aspects of the outcomes may support selective responding (Ostlund and Balleine 2007). Critically, our data also suggest that outcome-selective devaluation reduced responding not only in a selective but also in a non-selective manner, that is, reduced responding for the valued outcome. For instance, devaluation of pellets suppressed responding on the sucrose-predictive lever during presentation of the sucrose-predictive stimulus. Notably, similar effects were observed in a purely instrumental task, that is, post-training devaluation of pellets reduced lever pressing for pellets more than for sucrose (Colwill and Rescorla 1985). In view of the framework of sensory and value aspects underlying sPIT transfer effects, our data point to the possibility that not only sensory aspects of outcomes but also the value of outcomes could play a role in sPIT. Alternatively, as motivational downshifts used here consistently exert overall inhibitory effects on instrumental responding and approach behavior in a largely stimulus-independent manner, motivational downshifts may reduce general motivation, that is, blunt the motivation to engage in high rates of reward-directed behavior. This implies that general motivating effects may support sPIT performance. However, as noted above, subtle procedural details of the sPIT protocol being used could also contribute to suppress overall responding. For instance, it is conceivable that gradual variation of experimental procedures could result in a distinctive recruitment of behavioral mechanisms and neural circuits and, in turn, a distinctive impact of outcome value representations. Notably, testing for outcome devaluation sensitivity is also used to check whether instrumental responding is under goal-directed or habitual control. As with sPIT, multiple experimental procedures are used across studies, which, not surprisingly, may involve distinct behavioral and neural processes that control goal-directed vs habitual responding in slightly disparate ways, a fact that could account for in part discrepant results (Naneix et al., 2009; Lex and Hauber, 2010a; see Schreiner et al., 2020 for review).

Moreover, in the few available studies in humans, outcome devaluation had mixed effects on sPIT. One study demonstrated that sPIT was insensitive to devaluation of food-related outcomes (Watson et al., 2014), whereas others revealed that sPIT remained sensitive to devaluation of monetary-related outcomes (Allman et al., 2010; Eder and Dignath 2016). Again, methodological discrepancies including outcome identity may account for disparate results, at least to some extent.

Given the mixed data on outcome devaluation effects on sPIT, further research is required to substantiate the influential notion that food-predictive stimuli prevalent in environments may override satiety to promote overeating, obesity, and failures to change eating (Berthoud 2012; Petrovich 2013; Watson et al., 2014; Corbit and Balleine 2016). Our data based on sPIT suggest that, at least under the conditions tested here, satiety had relatively little influence on guidance of response selection by food-predictive stimuli, but very pronounced inhibitory effects on the overall level of reward-directed responding, thereby limiting food intake. Moreover, it is important to note that the magnitude of general inhibitory effects possibly also depends on how food-predictive stimuli and working for particular food rewards are learned and, in turn, which specific behavioral and neural mechanisms are involved.

## Interest Statement

There are no conflicts of interest for any of the authors.
